# 1,4-Dimethyl-3-phenyl-3*H*-pyrazolo[3,4-*c*]isoquinolin-5(4*H*)-one

**DOI:** 10.1107/S1600536808010180

**Published:** 2008-04-16

**Authors:** Fiorella Meneghetti, Gabriella Bombieri, Benedetta Maggio, Giuseppe Daidone

**Affiliations:** a Institute of Pharmaceutical and Toxicological Chemistry ‘P. Pratesi’, University of Milan, via L. Mangiagalli 25, 20133 Milan, Italy; b Department of Pharmaceutical and Technological Chemistry, University of Palermo, via Archirafi 32, 90123 Palermo, Italy

## Abstract

The title compound, C_18_H_15_N_3_O, is the product of the thermal decomposition of the diazo­nium salt derived from 2-amino-*N*-methyl-*N*-(3-methyl-1-phenyl-1*H*-pyrazol-5-yl)benzamide. It is characterized by a *trans* orientation of the methyl groups with respect to the tricyclic ring system. The mol­ecule has a nearly planar phenyl­pyrazolo[3,4-*c*]isoquinolin-5-one system, the largest deviation from the mean plane being 0.066 (2) Å for the O atom. The dihedral angle between the phenyl substituent and the heterotricycle is 67 (1)°. The packing is stabilized by C—H⋯N hydrogen-bond inter­actions, with the formation of mol­ecular chains along the *c* axis.

## Related literature

Pyrazole rings are useful templates to investigate the role of the aryl­diazo­nium group in the Pschorr reaction pathway (Maggio *et al.*, 2005 ▶). For related literature, see: Daidone *et al.* (1980 ▶, 1993 ▶, 1998 ▶).
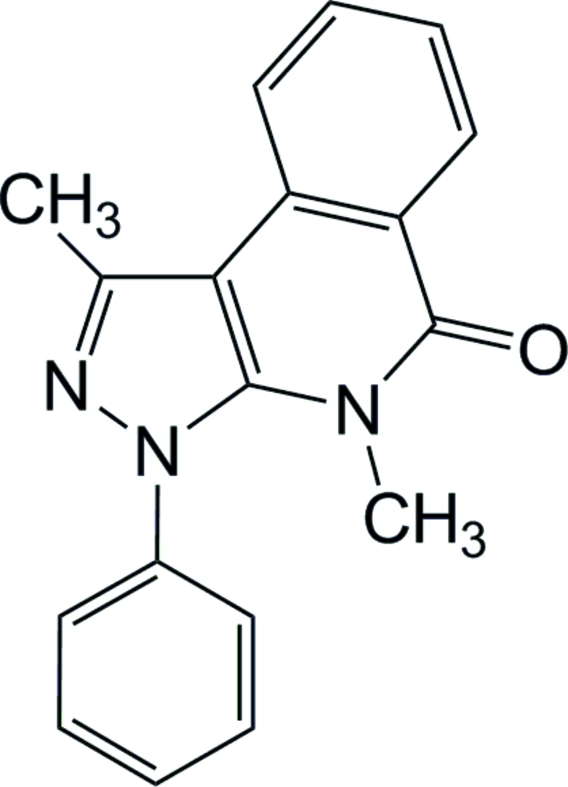


## Experimental

### 

#### Crystal data

C_18_H_15_N_3_O
*M* *_r_* = 289.33
Monoclinic, 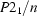

*a* = 8.066 (2) Å
*b* = 19.256 (3) Å
*c* = 9.270 (3) Å
β = 94.66 (3)°
*V* = 1435.0 (6) Å^3^
*Z* = 4
Mo *K*α radiation
μ = 0.09 mm^−1^
*T* = 293 (2) K
0.6 × 0.5 × 0.4 mm

#### Data collection

Enraf–Nonius TurboCAD-4 diffractometer
Absorption correction: none
2984 measured reflections
2815 independent reflections
1751 reflections with *I* > 2σ(*I*)
*R*_int_ = 0.031
3 standard reflections frequency: 120 min intensity decay: 3%

#### Refinement

*R*[*F* ^2^ > 2σ(*F* ^2^)] = 0.048
*wR*(*F* ^2^) = 0.113
*S* = 1.01
2815 reflections
202 parameters
H-atom parameters constrained
Δρ_max_ = 0.15 e Å^−3^
Δρ_min_ = −0.15 e Å^−3^

### 

Data collection: *CAD-4 EXPRESS* (Enraf–Nonius, 1994 ▶); cell refinement: *CAD-4 EXPRESS*; data reduction: *XCAD4* (Harms & Wocadlo, 1995 ▶); program(s) used to solve structure: *SIR92* (Altomare *et al.*, 1994 ▶); program(s) used to refine structure: *SHELXL97* (Sheldrick, 2008 ▶); molecular graphics: *ORTEP-3 for Windows* (Farrugia, 1997 ▶); software used to prepare material for publication: *WinGX* (Farrugia, 1999 ▶).

## Supplementary Material

Crystal structure: contains datablocks I, global. DOI: 10.1107/S1600536808010180/fj2107sup1.cif

Structure factors: contains datablocks I. DOI: 10.1107/S1600536808010180/fj2107Isup2.hkl

Additional supplementary materials:  crystallographic information; 3D view; checkCIF report

## Figures and Tables

**Table 1:** Hydrogen-bond geometry (Å, °)

*D*—H⋯*A*	*D*—H	H⋯*A*	*D*⋯*A*	*D*—H⋯*A*
C6—H6⋯N1^i^	0.93	2.59	3.457 (3)	156

Symmetry code: (i) 
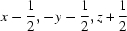
.

**Fig. 1.**
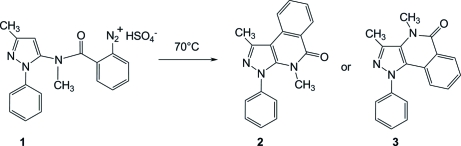
The chemical reaction scheme.

**Fig. 2.**
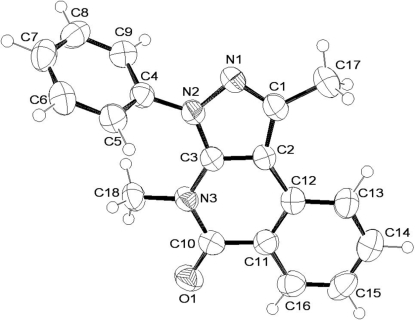
The molecular structure of the title compound, showing atom-labelling scheme. Displacement ellipsoids are drawn at the 50% probability level.

**Fig. 3.**
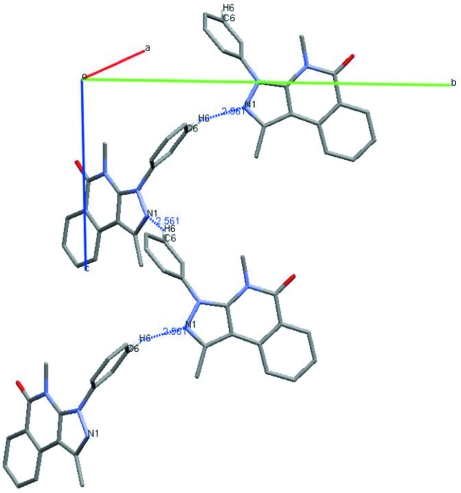
Intermolecular interactions of the title compound, showing the molecular chains along the c axis. Hydrogen bonds are shown as dashed lines.

## supplementary crystallographic information

### Comment

On the basis of our studies on the non classical Pschorr reaction (Maggio *et al.*, 2005), we have hypothesized that the product of thermal decomposition of the diazonium hydrogen sulfate (1) (Daidone *et al.*, 1980) could be one of the two possible isomers 2 and 3 (Fig. 1). Single-crystal X-ray analysis on the reaction product (Fig. 2) allows to assign the formation of isomer 2, having the two methyl groups *trans* oriented with respect to the tricyclic ring. The molecule is characterized by a quite planar phenylpyrazolo[3,4-*c*]isoquinolin-5-one moiety, having as highest deviation from planarity O1 atom (out of plane of 0.066 (2) Å). The non aromatic ring of the tricyclic framework has puckering parameter of φ2=-69.4 (2)° and QT=0.059 (3) Å. The phenyl substituent is inclined with respect to the heterotricycle of 67 (1)°, with a torsion angle N1—N2—C4—C5 of -106.1 (3)°. The molecular packing is determined by intermolecular C6—H6···N1i interactions of 2.56 (2)Å and 158 (1)° [symmetry code: (i) *x* - 1/2, -*y* - 1/2, *z* + 1/2], with the formation of chains developing along the *c* axis (Fig. 3).

### Experimental

The title compound was obtained as the product of the thermal decomposition of the diazonium salt derived from 2-amino-*N*-methyl-*N*-(3-methyl-1-phenyl-1*H*-pyrazol-5-yl)benzamide.

### Refinement

All non-H-atoms were refined anisotropically. Hydrogen atoms were introduced at calculated positions, in their described geometries and allowed to ride on the attached carbon atom with fixed isotropic thermal parameters (1.2U_eq_ and 1.5U_eq_ of the parent carbon atom for aromatic H-atoms and methyls H-atoms, respectively).

### Crystal data

**Table:**

C_18_H_15_N_3_O	*F*_000_ = 608
*M**_r_* = 289.33	*D*_x_ = 1.339 Mg m^−^^3^
Monoclinic, *P*2_1_/*n*	Mo *K*α radiation λ = 0.71073 Å
Hall symbol: -P 2yn	Cell parameters from 25 reflections
*a* = 8.066 (2) Å	θ = 9–10º
*b* = 19.256 (3) Å	µ = 0.09 mm^−^^1^
*c* = 9.270 (3) Å	*T* = 293 (2) K
β = 94.66 (3)º	Prism, colorless
*V* = 1435.0 (6) Å^3^	0.6 × 0.5 × 0.4 mm
*Z* = 4	

### Data collection

**Table:**

Enraf–Nonius TurboCAD-4 diffractometer	θ_max_ = 26.0º
Monochromator: graphite	θ_min_ = 3.1º
*T* = 293(2) K	*h* = −9→9
Non–profiled ω/2θ scans	*k* = 0→23
Absorption correction: none	*l* = 0→11
2984 measured reflections	3 standard reflections
2815 independent reflections	every 120 min
1751 reflections with *I* > 2σ(*I*)	intensity decay: 3%
*R*_int_ = 0.031	

### Refinement

**Table:**

Refinement on *F*^2^	Hydrogen site location: inferred from neighbouring sites
Least-squares matrix: full	H-atom parameters constrained
*R*[*F*^2^ > 2σ(*F*^2^)] = 0.048	*w* = 1/[σ^2^(*F*_o_^2^) + 1.3459*P*] where *P* = (*F*_o_^2^ + 2*F*_c_^2^)/3
*wR*(*F*^2^) = 0.113	(Δ/σ)_max_ = 0.006
*S* = 1.01	Δρ_max_ = 0.16 e Å^−^^3^
2815 reflections	Δρ_min_ = −0.15 e Å^−^^3^
202 parameters	Extinction correction: SHELXL97 (Sheldrick, 2008), Fc^*^=kFc[1+0.001xFc^2^λ^3^/sin(2θ)]^-1/4^
Primary atom site location: structure-invariant direct methods	Extinction coefficient: 0.043 (3)
Secondary atom site location: difference Fourier map	

### Special details

**Table:**

Geometry. All e.s.d.'s (except the e.s.d. in the dihedral angle between two l.s. planes) are estimated using the full covariance matrix. The cell e.s.d.'s are taken into account individually in the estimation of e.s.d.'s in distances, angles and torsion angles; correlations between e.s.d.'s in cell parameters are only used when they are defined by crystal symmetry. An approximate (isotropic) treatment of cell e.s.d.'s is used for estimating e.s.d.'s involving l.s. planes.
Refinement. Refinement of *F*^2^ against ALL reflections. The weighted *R*-factor *wR* and goodness of fit *S* are based on *F*^2^, conventional *R*-factors *R* are based on *F*, with *F* set to zero for negative *F*^2^. The threshold expression of *F*^2^ > σ(*F*^2^) is used only for calculating *R*-factors(gt) *etc*. and is not relevant to the choice of reflections for refinement. *R*-factors based on *F*^2^ are statistically about twice as large as those based on *F*, and *R*- factors based on ALL data will be even larger.

### Fractional atomic coordinates and isotropic or equivalent isotropic displacement parameters (Å^2^)

**Table:**

	*x*	*y*	*z*	*U*_iso_*/*U*_eq_	
O1	0.62196 (18)	0.08361 (7)	0.50784 (16)	0.0628 (4)	
N1	0.93284 (19)	−0.15752 (8)	0.28089 (16)	0.0508 (4)	
N2	0.89261 (18)	−0.12641 (8)	0.40852 (16)	0.0447 (4)	
C1	0.8813 (2)	−0.11483 (10)	0.1753 (2)	0.0470 (5)	
C2	0.8049 (2)	−0.05543 (9)	0.22995 (19)	0.0415 (4)	
C3	0.8148 (2)	−0.06473 (9)	0.37789 (19)	0.0395 (4)	
C4	0.9007 (2)	−0.17029 (9)	0.53322 (19)	0.0417 (4)	
C5	0.7563 (2)	−0.19339 (10)	0.5872 (2)	0.0504 (5)	
H5	0.6535	−0.1779	0.5469	0.060*	
C6	0.7650 (3)	−0.23975 (11)	0.7016 (2)	0.0571 (6)	
H6	0.6680	−0.2551	0.7391	0.068*	
C7	0.9167 (3)	−0.26301 (10)	0.7598 (2)	0.0586 (6)	
H7	0.9222	−0.2948	0.8355	0.070*	
C8	1.0615 (3)	−0.23927 (10)	0.7063 (2)	0.0592 (6)	
H8	1.1642	−0.2545	0.7471	0.071*	
C9	1.0537 (2)	−0.19281 (10)	0.5918 (2)	0.0496 (5)	
H9	1.1506	−0.1770	0.5549	0.060*	
N3	0.76230 (18)	−0.01656 (8)	0.47488 (16)	0.0427 (4)	
C10	0.6777 (2)	0.04286 (10)	0.4218 (2)	0.0460 (5)	
C11	0.6673 (2)	0.05442 (9)	0.2651 (2)	0.0458 (5)	
C12	0.7305 (2)	0.00692 (10)	0.1681 (2)	0.0446 (5)	
C13	0.7159 (3)	0.02280 (11)	0.0198 (2)	0.0581 (6)	
H13	0.7562	−0.0083	−0.0457	0.070*	
C14	0.6434 (3)	0.08330 (12)	−0.0299 (3)	0.0681 (6)	
H14	0.6348	0.0929	−0.1286	0.082*	
C15	0.5829 (3)	0.13036 (12)	0.0653 (3)	0.0665 (6)	
H15	0.5345	0.1716	0.0309	0.080*	
C16	0.5943 (2)	0.11612 (11)	0.2113 (2)	0.0586 (6)	
H16	0.5530	0.1479	0.2749	0.070*	
C18	0.7939 (3)	−0.02364 (10)	0.63194 (19)	0.0529 (5)	
H18A	0.7052	−0.0496	0.6694	0.079*	
H18B	0.8000	0.0216	0.6756	0.079*	
H18C	0.8972	−0.0476	0.6539	0.079*	
C17	0.9067 (3)	−0.13436 (12)	0.0218 (2)	0.0668 (6)	
H17A	0.9664	−0.1775	0.0208	0.100*	
H17B	0.9694	−0.0987	−0.0214	0.100*	
H17C	0.8006	−0.1394	−0.0320	0.100*	

### Atomic displacement parameters (Å^2^)

**Table:**

	*U*^11^	*U*^22^	*U*^33^	*U*^12^	*U*^13^	*U*^23^
O1	0.0762 (10)	0.0510 (9)	0.0636 (10)	0.0104 (7)	0.0201 (8)	−0.0068 (7)
N1	0.0585 (10)	0.0518 (10)	0.0430 (10)	0.0078 (8)	0.0096 (8)	−0.0067 (8)
N2	0.0528 (10)	0.0429 (9)	0.0388 (9)	0.0041 (7)	0.0070 (7)	−0.0005 (7)
C1	0.0490 (11)	0.0520 (11)	0.0405 (11)	−0.0016 (9)	0.0060 (8)	−0.0056 (10)
C2	0.0444 (10)	0.0429 (11)	0.0374 (10)	−0.0027 (8)	0.0055 (8)	−0.0014 (9)
C3	0.0380 (10)	0.0400 (10)	0.0409 (11)	−0.0038 (8)	0.0046 (8)	−0.0025 (9)
C4	0.0468 (11)	0.0382 (10)	0.0402 (11)	−0.0012 (8)	0.0030 (8)	−0.0032 (8)
C5	0.0464 (11)	0.0561 (12)	0.0484 (12)	−0.0026 (10)	0.0030 (9)	−0.0014 (10)
C6	0.0665 (14)	0.0578 (13)	0.0481 (12)	−0.0148 (11)	0.0118 (11)	−0.0022 (11)
C7	0.0893 (17)	0.0431 (12)	0.0426 (12)	−0.0036 (11)	0.0014 (11)	0.0009 (9)
C8	0.0641 (14)	0.0507 (13)	0.0600 (14)	0.0091 (10)	−0.0116 (11)	−0.0007 (11)
C9	0.0482 (11)	0.0452 (11)	0.0549 (12)	−0.0022 (9)	0.0007 (9)	−0.0025 (10)
N3	0.0488 (9)	0.0420 (9)	0.0380 (9)	−0.0013 (7)	0.0075 (7)	−0.0034 (7)
C10	0.0455 (11)	0.0400 (11)	0.0534 (12)	−0.0023 (9)	0.0094 (9)	−0.0030 (9)
C11	0.0437 (10)	0.0436 (11)	0.0501 (12)	−0.0026 (9)	0.0048 (9)	0.0044 (10)
C12	0.0438 (11)	0.0467 (11)	0.0433 (11)	−0.0080 (9)	0.0024 (9)	0.0015 (9)
C13	0.0686 (14)	0.0578 (14)	0.0480 (13)	−0.0017 (11)	0.0056 (10)	0.0050 (11)
C14	0.0792 (16)	0.0678 (15)	0.0565 (15)	−0.0022 (13)	0.0006 (12)	0.0168 (12)
C15	0.0652 (14)	0.0598 (14)	0.0735 (16)	0.0044 (11)	0.0000 (12)	0.0256 (13)
C16	0.0551 (13)	0.0503 (12)	0.0713 (15)	0.0030 (10)	0.0098 (11)	0.0067 (11)
C18	0.0676 (13)	0.0530 (12)	0.0386 (11)	−0.0022 (10)	0.0076 (9)	−0.0058 (9)
C17	0.0786 (15)	0.0777 (16)	0.0449 (13)	0.0079 (12)	0.0099 (11)	−0.0136 (11)

### Geometric parameters (Å, °)

**Table:**

O1—C10	1.230 (2)	C9—H9	0.9300
N1—C1	1.319 (2)	N3—C10	1.401 (2)
N1—N2	1.3879 (19)	N3—C18	1.464 (2)
N2—C3	1.362 (2)	C10—C11	1.466 (3)
N2—C4	1.429 (2)	C11—C16	1.400 (3)
C1—C2	1.412 (2)	C11—C12	1.407 (2)
C1—C17	1.502 (2)	C12—C13	1.404 (3)
C2—C3	1.379 (2)	C13—C14	1.366 (3)
C2—C12	1.440 (3)	C13—H13	0.9300
C3—N3	1.382 (2)	C14—C15	1.382 (3)
C4—C9	1.377 (2)	C14—H14	0.9300
C4—C5	1.378 (2)	C15—C16	1.376 (3)
C5—C6	1.384 (3)	C15—H15	0.9300
C5—H5	0.9300	C16—H16	0.9300
C6—C7	1.371 (3)	C18—H18A	0.9600
C6—H6	0.9300	C18—H18B	0.9600
C7—C8	1.383 (3)	C18—H18C	0.9600
C7—H7	0.9300	C17—H17A	0.9600
C8—C9	1.385 (3)	C17—H17B	0.9600
C8—H8	0.9300	C17—H17C	0.9600
			
C1—N1—N2	106.38 (15)	O1—C10—N3	119.15 (18)
C3—N2—N1	109.53 (14)	O1—C10—C11	123.45 (18)
C3—N2—C4	132.37 (15)	N3—C10—C11	117.34 (17)
N1—N2—C4	115.84 (14)	C16—C11—C12	119.19 (18)
N1—C1—C2	111.02 (16)	C16—C11—C10	118.10 (18)
N1—C1—C17	119.19 (17)	C12—C11—C10	122.70 (17)
C2—C1—C17	129.78 (18)	C13—C12—C11	118.58 (18)
C3—C2—C1	105.07 (16)	C13—C12—C2	124.78 (18)
C3—C2—C12	119.52 (17)	C11—C12—C2	116.64 (17)
C1—C2—C12	135.39 (17)	C14—C13—C12	121.0 (2)
N2—C3—C2	107.99 (16)	C14—C13—H13	119.5
N2—C3—N3	127.58 (16)	C12—C13—H13	119.5
C2—C3—N3	124.33 (17)	C13—C14—C15	120.5 (2)
C9—C4—C5	120.77 (17)	C13—C14—H14	119.8
C9—C4—N2	119.08 (16)	C15—C14—H14	119.8
C5—C4—N2	119.99 (16)	C16—C15—C14	119.9 (2)
C4—C5—C6	119.64 (19)	C16—C15—H15	120.0
C4—C5—H5	120.2	C14—C15—H15	120.0
C6—C5—H5	120.2	C15—C16—C11	120.8 (2)
C7—C6—C5	120.03 (19)	C15—C16—H16	119.6
C7—C6—H6	120.0	C11—C16—H16	119.6
C5—C6—H6	120.0	N3—C18—H18A	109.5
C6—C7—C8	120.2 (2)	N3—C18—H18B	109.5
C6—C7—H7	119.9	H18A—C18—H18B	109.5
C8—C7—H7	119.9	N3—C18—H18C	109.5
C7—C8—C9	120.1 (2)	H18A—C18—H18C	109.5
C7—C8—H8	120.0	H18B—C18—H18C	109.5
C9—C8—H8	120.0	C1—C17—H17A	109.5
C4—C9—C8	119.28 (19)	C1—C17—H17B	109.5
C4—C9—H9	120.4	H17A—C17—H17B	109.5
C8—C9—H9	120.4	C1—C17—H17C	109.5
C3—N3—C10	119.09 (16)	H17A—C17—H17C	109.5
C3—N3—C18	123.15 (16)	H17B—C17—H17C	109.5
C10—N3—C18	117.75 (15)		

### Hydrogen-bond geometry (Å, °)

**Table:**

*D*—H···*A*	*D*—H	H···*A*	*D*···*A*	*D*—H···*A*
C6—H6···N1^i^	0.93	2.59	3.457 (3)	156

Symmetry codes: (i) *x*−1/2, −*y*−1/2, *z*+1/2.
